# Amniotic Fluid and Maternal Serum Laeverin Levels and Their Correlations with Fetal Size and Placental Volume in Second Trimester of Pregnancy—A Prospective Cross-Sectional Study

**DOI:** 10.3390/diagnostics15030317

**Published:** 2025-01-29

**Authors:** János Sikovanyecz, Giuseppe Gullo, Márió Vincze, Imre Földesi, Gábor Németh, Andrea Surányi, János Sikovanyecz, Zoltan Kozinszky

**Affiliations:** 1 Department of Obstetrics and Gynecology, University of Szeged, 6725 Szeged, Hungary; janossikovanyecz@gmail.com (J.S.J.); vincze.mario92@gmail.com (M.V.); nemeth.gabor@med.u-szeged.hu (G.N.); gaspar-suranyi.andrea@med.u-szeged.hu (A.S.); drsikovanyecz@gmail.com (J.S.); 2 IVF Unit, Department of Obstetrics and Gynecology, Villa Sofia Cervello Hospital, University of Palermo, 90146 Palermo, Italy; gullogiuseppe@libero.it; 3 Department of Laboratory Medicine, University of Szeged, 6720 Szeged, Hungary; foldesi.imre@med.u-szeged.hu; 4 Capio Specialized Center for Gynecology Solna, 182 88 Stockholm, Sweden

**Keywords:** human laeverin, serum, amniotic fluid, fetus, placenta, ultrasound

## Abstract

**Background:** Laeverin is an extravillous trophoblast marker playing a significant role in trophoblast migration. We endeavored to estimate the association between the amniotic and serum laeverin concentrations at 16–22 weeks of gestation and the fetal and placental ultrasound measurements in high-risk uncomplicated pregnancies. **Methods:** A prospective cross-sectional study of consecutively recruited singleton pregnancies undergoing amniocentesis was performed. Fetal structural malformations and/or aneuploidy were the exclusion criteria. Fetal biometric parameters and placental growth/perfusion were assessed by ultrasound in 44 high-risk pregnancies who had no pregnancy complications and any other chronic disease. Maternal serum and amniotic laeverin levels were essayed with sandwich enzyme-linked immunosorbent assay. **Results:** Serum laeverin levels are decreasing marginally with the maternal age in mid-gestation. Laeverin levels in the serum correlated minimally negatively with head size of the fetus (β = −0.38; *p* < 0.05; 95% confidence interval (CI) −0.03–0.01), whereas the amniotic level correlated strongly with the fetal abdominal circumference (β = −0.74; *p* < 0.05; 95% CI: −0.34–−0.09). In addition, the amniotic laeverin level correlated moderately and positively with the placental volume (β = 0.46; *p* < 0.05; 95% CI: 0.01–0.08). **Conclusions:** Laeverin levels detected in the serum and in the amniotic fluid denote the fetoplacental growth in uncomplicated high-risk pregnancies.

## 1. Introduction

Aminopeptidase-Q, often referred to as laeverin, is secreted by the extravillous trophoblast (EVT) and plays a crucial role in EVT invasion into the maternal decidua/myometrium, as well as their migration into the spiral arteries. It plays a key regulatory role in the epithelial-to-mesenchymal transition of EVTs and the remodeling of the spiral arteries within the uterine wall [1,2,3]. Laeverin is as an aminopeptidase localized on the cell membrane of EVTs, interacting with the maternal immune system [4,5,6]. It can cleave various substrates, including anti-migratory oligopeptides like kisspeptin-10, and several angiogenesis-related molecules such as angiotensin III, endokinin-C, and dynorphin A1-8. These angiogenic substances are crucial for regulating local blood pressure and ensuring adequate blood flow to the placenta, particularly in the placental bed [7].

Laeverin plays a significant role in the molecular network that regulates placental growth, facilitating EVT migratory capabilities through the regulated degradation of the extracellular matrix in the maternal decidua, mediated by the synchronization of matrix metalloproteinases and their tissue inhibitors [8]. Additionally, laeverin collaborates with the chemokine inflammatory system at the fetomaternal interface to maintain proper angiogenesis in the placenta [9]. It down-regulates N-cadherin and vimentin while promoting the production of E-cadherin, which facilitates the movement of decidual EVTs. Laeverin also converts kallikrein into bradykinin, which maintains a pro-inflammatory environment in the placenta and enhances EVT migration [3,10,11]. Nonetheless, direct molecular evidence is lacking, as the enzymatic functions of laeverin were elucidated through molecular experiments utilizing functional physiological analogies to describe its characteristics [4]. Moreover, research on trophoblast invasion and changes in enzymatic functions during interactions with the uterine environment, conducted in trophoblast assays and explant tissues, tends to have limited significance [12].

Silencing laeverin in trophoblast migration studies suggests that it regulates EVT outgrowth [4,5,6,13]. Some studies indicate the potential role of laeverin in the pathogenesis of the impaired placental function in preeclampsia. The increased laeverin production is associated with deterred EVT migration, leading to a shallow placentation and poor remodeling of spiral arteries. In cases of preeclampsia, maternal serum laeverin levels in the third trimester are lower compared to uncomplicated pregnancies, which is explained by the laeverin being entrapped in subcellular levels in the placenta [4,5,6,14]. Laeverin can be detected in placental tissue from the fourth week of gestation until term [5,6,15], indicating its involvement in non-EVT-related placental homeostasis molecular processes as EVT migration finishes by the 16th week of gestation [7]. Notably, laeverin levels of serum are high in the first trimester [16] but decrease by the third trimester [14]. Our research group previously noted that while laeverin is present in the amniotic fluid of high-risk pregnancies, its concentration does not predict the onset of preeclampsia. Although levels in amniotic fluid are higher than in serum, the role of laeverin in fetal life or the amniotic cavity remains unclear [17].

The amniotic laeverin levels were not determined in uncomplicated pregnancies in the mid-trimester. We sought to investigate the levels of laeverin in amniotic fluid during uncomplicated pregnancies and analyze its relationship with fetal weight, placental volume, and perfusion as measured by ultrasound.

## 2. Materials and Methods

### 2.1. Study Design and Population

A prospective, cross-sectional cohort study was conducted among pregnant women presented for amniocentesis at the Department of Obstetrics and Gynecology, University of Szeged, Hungary, from May 2022 to January 2023. During this study period, all singleton pregnancies at high-risk of chromosomal abnormalities who underwent amniocentesis between 16 + 0 and 22 + 6 weeks of gestation were consecutively enrolled. The exclusion criteria included the following: multiple pregnancies; fetal or neonatal structural and/or genetic anomalies; pathological placentation (such as placenta accreta spectrum or placenta previa); improper placentation for sonographic measurement of volume and perfusion (placenta on the posterior wall); self-reported substance abuse (drugs, alcohol, caffeine, nicotine); and systemic diseases (e.g., any type of pregestational diabetes mellitus, autoimmune diseases, vasculitis, hemophilia, thrombophilia, chronic infections). Pregnancies complicated later by gestational diabetes (diet/insulin treated, *n* = 11), hypertension-related diseases (*n* = 10), fetal growth restriction at delivery (*n* = 4), premature rupture of membranes or premature delivery (*n* = 6), or gestational age at birth (*n* = 9) were also excluded. Clinical follow-up and outcome data were obtained from the medical charts.

### 2.2. Ethics Statements

All participants received an oral and written explanation of the study, and informed consent was obtained from those who agreed to take part. The Clinical Research Ethics Committee of the University of Szeged approved the study protocol (reference number: 09/2017, approved on 10 February 2017). The study adhered to the principles of the Declaration of Helsinki and its amendments.

### 2.3. Conventional 2-Dimensional (2-D) and 3D Sonographic Examinations

The ultrasound methods utilized in prenatal examinations were previously described [17]. Briefly, conventional 2D sonographic techniques were initially employed, with pregnancies dated using crown-rump length (CRL) measurements during nuchal screening at 11 to 13 weeks of gestation. This assessment included evaluations of fetal anatomy, biometry, anomalies, placental location, and amniotic fluid volume. Estimations of fetal weight (EFW) were conducted using Hadlock’s method and compared to local standards, with EFW percentiles based on Hungarian nomograms [18]. The EFW percentile was presented based on the Hungarian nomograms [19].

Additionally, the study used the acquisition of placental volume images and three-dimensional power Doppler (3-DPD) indices through a Voluson 730 Expert ultrasound machine (GE Medical Systems, Kretztechnik GmbH & Co OHG, Tiefenbach, Austria), focusing on areas with the highest vascular density in the villi below the insertion of the umbilical cord. Images were processed in 3D rendering mode and later analyzed with the virtual organ computer-aided analysis (VOCAL) program [20]. The power Doppler indices—Vascularization Index (VI), Flow Index (FI), and Vascularization Flow Index (VFI)—were determined to quantify blood flow within the volume, providing estimates of blood vessel proportion, average power Doppler amplitude, and the product of these values. Excellent intra-observer reliability for these indices was confirmed, with intra-class correlation coefficients of 0.99 [21,22].

### 2.4. Amniocentesis Procedure

Patients were thoroughly informed about the amniocentesis procedure and its potential risks before consenting to participate. Each procedure was carried out by a single experienced operator (J.S.) following standard protocols. Local antiseptic was employed, and a 22-gauge spinal needle was used under constant ultrasound control, ensuring no entry across the placenta. Approximately 8–10 mL of amniotic fluid was aspirated, discarding the first 2 mL to avoid contamination with maternal cells. Amniotic fluid with blood was not used for the study preparation. Fetal heart rates were monitored after amniocentesis to verify viability, and no stillbirths or premature ruptures were documented. If necessary, anti-D immunoglobulin was given, and participants were advised to rest for 4–6 h following the procedure.

### 2.5. Samples

At the time of amniocentesis, approximately 5 mL of amniotic fluid and 10 mL of venous blood of each mother who participated in the study were analyzed. The blood samples were centrifuged at 3400× *g* rpm for 15 min, and both samples of serum and amniotic fluid were kept at −80 °C until analysis.

### 2.6. Enzyme-Linked Immunosorbent Assay (ELISA)

Laeverin concentrations were measured in maternal serum and amniotic fluid using a sandwich ELISA kit (Mybiosource, San Diego, CA, USA; catalog number: MBS2882930). Laboratory staff conducting the assays were blinded to the outcome of the pregnancy, and clinicians involved in recruitment did not analyze the samples. The sensitivity of laeverin assay was 0.23 ng/mL, with an intra-assay coefficient of ≤4.7% and an inter-assay coefficient of ≤6.3%, as reported by the manufacturer.

### 2.7. Data and Statistical Analysis

Statistics were computed and calculated employing SPSS version 23 (IBM Corp., Armonk, NY, USA). Continuous characteristics were presented as mean ± standard deviation (SD), while categorical variables were displayed as numbers and percentages. The relationship between continuous variables and laeverin levels was evaluated applying Pearson’s correlation and linear regression. Univariate and multivariate regression analyses were utilized to evaluate the relationship between laeverin levels and continuous variables, interpreting the results via correlation coefficients (β) and 95% confidence intervals (CIs). Multivariate linear regression is customized for standard confounders, including age, BMI at the time of amniocentesis, preceding gestations, and gestational length at amniocentesis, which can influence fetal weight and placental volume. Statistical significance was presented by a *p*-value < 0.05. Holm–Bonferroni adjustment was used for multiple comparisons.

## 3. Results

The most frequent reasons for amniocentesis include advanced maternal age (Table 1). Previous pregnancies were affected by aneuploidy in 15% of cases. Maternal characteristics and perinatal outcomes are summarized in Table 2. Most participants presented for amniocentesis were of advanced maternal age (>37 y), with approximately one-third being primiparous. The gestational age at amniocentesis ranged from 16 + 0 to 22 + 0 weeks. Neonatal weight and gestational age at birth showed no extremities since large and small for gestational age neonates were excluded from the study. The fetal sonographic parameters were consistent with national data [19], while placental volume and perfusion measurements were different from those of uncomplicated high-risk pregnancies published by our research group earlier before (Table 3) [23]. Notably, laeverin concentration was significantly elevated in amniotic fluid compared to maternal serum (Table 4).

**Figure 1:**
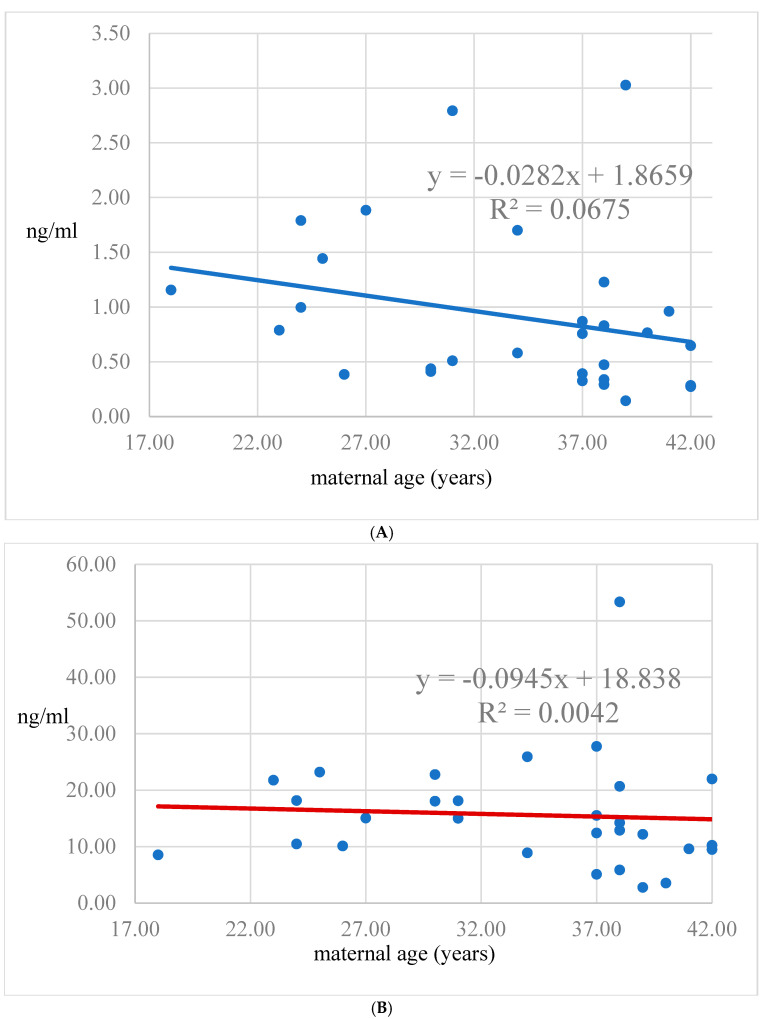
(**A**). Laeverin levels in serum plotted against maternal age. (**B**) Laeverin levels in amnion plotted against maternal age.

**Figure 2:**
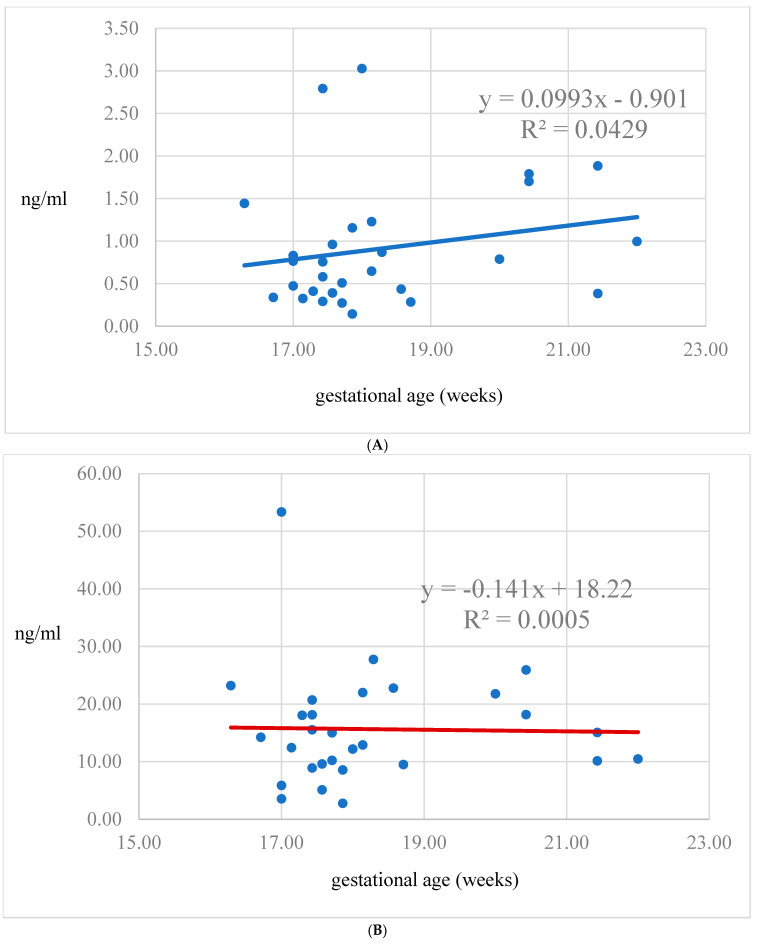
(**A**). Laeverin levels in serum plotted against gestational age. (**B**). Laeverin levels in serum plotted against gestational age.

A significant negative correlation was found between serum laeverin levels and maternal age (*p* < 0.05, β = −0.38, 95% CI = −0.07 to −0.01) (Figure 1A), whereas the corresponding figure was not significant in the amniotic fluid (Figure 1B). The laeverin concentration in the serum exhibits a non-significant rise during mid-gestation (Figure 2A), while the laeverin concentration in the amnion remains stable throughout this period (Figure 2B). Among the ultrasound parameters, fetal head circumference displayed a negative correlation with laeverin levels (*p* < 0.05, β = −0.38, 95% CI = −0.03 to −0.01). An inverse correlation was noted between abdominal circumference (both absolute values *p* < 0.05, β = −1.40, 95% CI = −1.15 to 0.02 and expressed in percentiles *p* < 0.05, β = −0.64, 95% CI = −0.31 to 0.06) and amniotic laeverin levels. A positive correlation was observed, indicating that higher placental volume was associated with increased laeverin levels (*p* < 0.05, β = 0.46, 95% CI = 0.01 to 0.08) (Table 5).

## 4. Discussion

Laeverin was detectable in amniotic fluid, which aligns with our previous finding [17]. The source of laeverin production in the amniotic fluid remains uncertain, as no molecular evidence through immunostaining confirms its production from amniotic epithelial cell membranes in late pregnancy [1]. Given that laeverin is a glycosylated protein enzyme, it is unlikely to diffuse through the subamniotic layers (mesenchymal stroma and basement membrane) [24]. One possible explanation is that laeverin is expressed by the amniotic membrane cells during the mid-trimester, although its secretion remains constant throughout this period. Another possibility is that the fetus secretes laeverin into the amniotic cavity as the fluid surrounds the fetus. However, this is somewhat contradicted by the observation that laeverin levels in the umbilical vessels (artery vs. vein) after birth are similar [14], suggesting that laeverin may not have a distinct role during fetal life prior to delivery. In contrast, the consistently high concentration of laeverin in the amnion observed in our study may suggest its crucial role in fetal development. The only indirect suggestion so far that laeverin may have a role in fetal circulation under certain pathological conditions is that an increased level of laeverin in the fetal circulation could be expected in preeclampsia, as leakage of the protein into the fetal capillaries in the chorionic villi was described in preeclamptic placentas collected after delivery [5]. Notably, serum laeverin levels increase during the gestational period examined in our study, which coincides with the sprouting of EVTs into the decidua, the inner third of the myometrium, and the spiral arteries. One might speculate that the rising laeverin concentration in the maternal serum reflects the trafficking activity of the EVTs.

The levels of laeverin in amniotic fluid seem linked to placental development, volume, and fetal growth. This may be due to an increase in chorionic mass—including both extra- and intravillous trophoblasts—as gestation progresses, leading to greater laeverin production [1,5,14].

Laeverin levels are particularly high in amniotic fluid between 16 and 22 weeks of gestation, while significantly lower levels (by one order of magnitude) are found in serum. This finding is consistent with our past studies demonstrating higher laeverin excretion into the amniotic cavity during mid-gestation, regardless of pregnancy complication [17]. Notably, serum laeverin levels show a slight increase during the first trimester (8–14 weeks) [16] and a further increasing trend in the second trimester can be detected to the mid-pregnancy, as described in the current study and in our previous study [17]. However, a sharp decrease in the latter half of pregnancy [14] can be noted, coinciding with the placental function and the completion of spiral artery remodeling [25]. However, large variations in laeverin levels reported in different studies could be attributed to sampling methods and the commercial ELISA kit types (direct vs. non-direct, sandwich, competitive, etc.) used for measurements [14,16]. However, the sandwich ELISA kit is an appropriate test for the quantitation of soluble laeverin homodimer according to the manufacturer. Following delivery, laeverin levels in maternal serum decrease sharply [14].

Interestingly, while laeverin production in the placenta typically increases as gestation progresses in normal pregnancies [4,6,16], the levels of laeverin detected in maternal serum decline [14]. This pattern suggests that laeverin may have a vital role in placental homeostasis, particularly in the first half of pregnancy [15], given that EVT migration finishes by around 16 weeks gestation [26]. This was also confirmed by our results, which demonstrate a direct relationship between amniotic laeverin concentration and placental volume as measured by ultrasound, highlighting the need for further molecular examination of the placenta from the second half of pregnancy. Laeverin is a stable, cell-membrane-bound protein with a slow turnover, playing a crucial role in EVT migration into the uterine wall [2,3,4,5,6,13,27]. It is specifically expressed in EVTs throughout pregnancy, co-expressing with HLA-G as a marker of EVT activity [13].

There is very limited evidence suggesting that laeverin could serve as a serum biomarker for preeclampsia. In cases of preeclampsia, there is often an overexpression of laeverin in the placenta, potentially leading to a significant difference in serum levels during the early third trimester [5,6,14]. The enhanced intracellular effects of laeverin could suppress the migratory abilities of EVTs, which is characteristic of preeclampsia [4,5,6]. Previous studies found no significant correlation between early serum laeverin levels and the differentiation between preeclampsia and unaffected controls during the first trimester [16]. In our prior research, we did not find a meaningful relationship between laeverin levels in serum or amniotic fluid and the later development of preeclampsia [28]. Nevertheless, there were very few participants with hypertensive conditions in both studies, which may limit generalizability [16,17]. Conversely, some evidence suggests that lower serum laeverin levels at 22–24 weeks could indicate an increased risk of developing preeclampsia later [14].

Our study has its limitations due to the small number of samples and the purified group for maternal age, as a main influencing factor of laeverin levels. Additionally, our findings indicate that serum laeverin levels decrease with maternal age. Given that advanced maternal age is associated with reduced placental volumetric measurements [23], it is plausible that lower levels of EVT lead to reduced laeverin secretion. Interestingly, while amniotic laeverin levels correlate well with fetal abdominal diameter, serum laeverin levels are more closely related to fetal head circumference, and both parameters significantly determine actual fetal weight. Further investigations are necessary to elucidate the reasons behind the association between laeverin levels in various body fluids and fetal parameters. It seems evident that larger fetuses have correspondingly larger placentas, reflecting the extent of laeverin secretion. Our previous research indicated a slight to moderate correlation between laeverin levels and both fetal and placental sizes, particularly in complicated pregnancies [17]. However, in this study, fetal growth parameters exhibited an opposite correlation with laeverin levels in uncomplicated pregnancies, warranting further exploration into how laeverin influences fetal growth across different pregnancy conditions.

## 5. Conclusions

This study demonstrated that laeverin can be found in the amniotic fluid of uncomplicated, high-risk pregnancies, marking its potential physiological role in the amniotic cavity. Serum laeverin levels increase, while amniotic fluid laeverin levels remain constant during mid-gestation. Furthermore, serum laeverin concentration decreases with increasing maternal age, whereas amniotic fluid laeverin levels show no change with advancing maternal age. Furthermore, laeverin levels measured in these body fluids show mild to moderate correlations with estimated placental volume and fetal weight parameters. Further research is necessary to clarify the functional role of laeverin in fetoplacental well-being and its implications for both fetal and placental health in various pregnancy conditions.

## Figures and Tables

**Table 1:** Indication of amniocentesis.

Indications for Amniocentesis	Number of Cases
increased nuchal translucency (NT) at first trimester scan (≥2 MoM for gestational age)	12
previous pregnancy affected by a chromosomal or genetic abnormality	7
advanced maternal age (>37 y)	25

**Table 2:** Clinical and obstetrical data of women with amniocentesis (*n* = 44).

Maternal age (years) *	33.59 ± 6.49
Number of nulliparous women in the study **	13 (29.5)
BMI at the time of genetical consultation (kg/m^2^) *	26.42 ± 5.80
Gestational age at the time of amniocentesis (weeks) *	18.30 ± 1.44
Birthweight (grams) *	3519.55 ± 440.76
Gestational age at delivery (weeks) *	38.99 ± 0.99

* Continuous variables displayed as mean ± standard deviation (SD). ** Categorical variables are presented in number and %.

**Table 3:** Ultrasound data at amniocentesis (*n* = 44) *.

Fetal Biometry	
Head circumference (mm)	152.79 ± 16.08
Head circumference (percentile)	53.09 ± 30.32
Abdominal circumference (mm)	130.51 ± 16.30
Abdominal circumference (percentile)	49.94 ± 29.83
Femur length (mm)	27.53 ± 4.83
Femur length (percentile)	58.75 ± 30.15
Estimated birthweight (grams)	251.48 ± 75.68
Estimated birthweight (percentile)	52.20 ± 25.87
Placental sonography	
Placental volume (mm^3^)	231.58 ± 95.01
VI	14.57 ± 5.19
FI	48.69 ± 27.29
VFI	8.08 ± 3.70

VI: Vascularization Index; FI: Flow Index; VFI: Vascularization Flow Index. * Continuous variables displayed as mean ± standard deviation (SD).

**Table 4:** Levels of angiogenic factors in samples of amniotic fluid and serum (*n* = 44) *.

Laeverin in amniotic fluid (ng/mL)	15.64 ± 9.84
Laeverin concentration in serum (ng/mL)	0.91 ± 0.73

* Continuous variables displayed as mean ± standard deviation (SD).

**Table 5:** Correlation between maternal as well as sonographic data and levels of laeverin in maternal serum and amniotic fluid (*n* = 44).

Variables	Laeverin Level in the Serum				Laeverin Level in the Amniotic Fluid			
	Simple Linear Regression		Multivariate Linear Regression		Simple Linear Regression		Multivariate Linear Regression	
	β	95% CI	β	95% CI	β	95% CI	β	95% CI
Maternal characteristics								
Maternal age	−0.38 *	−0.07–0.01 *	−0.23	−0.08–0.03	−0.06	−0.67–0.48	−0.08	−0.90–0.67
Previous parity	−0.04	−0.33–0.27	0.09	−0.28–0.42	−0.02	−4.21–3.88	0.01	−4.83–4.87
BMI at the time of genetical consultation (kg/m^2^)	−0.17	−0.07–0.03	−0.08	−0.06–0.04	−0.08	−0.78–0.52	−0.07	−0.85–0.61
Birthweight (grams)	0.10	0.01–0.01	0.20	0.01–0.01	−0.03	−0.01–0.01	−0.02	−0.01–0.01
Birthweight (percentile)	−0.05	−0.01–0.01	0.04	−0.01–0.01	−0.11	−0.19–0.11	−0.10	−0.21–0.14
GA at delivery	0.36	−0.01–0.08	0.41	0.01–0.08	0.11	−0.41–0.71	0.11	−0.46–0.77
GA at the time of amniocentesis (weeks)	0.21	−0.01–0.04	0.10	−0.03–0.04	−0.02	−0.39–0.34	−0.08	−0.52–0.38
Ultrasound characteristics								
Head circumference (mm)	−0.38 *	−0.03–0.01 *	−0.48	−0.04–−0.03	−0.09	−0.30–0.19	−0.16	−0.39–0.20
Head circumference (percentile)	−0.20	−0.02–0.01	−0.25	−0.02–0.01	0.03	−0.13–0.15	0.12	−0.14–0.22
Abdominal circumference (mm)	−0.01	−0.03–0.03	−0.84	−0.14–0.06	0.23	−0.17–0.35	−1.40 *	−1.15–0.02 *
Abdominal circumference (percentile)	−0.31	−0.03–0.01	−0.29	−0.04–0.02	−0.74 **	−0.34–−0.09 **	−0.64 *	−0.31–0.06 *
Femur length (mm)	0.17	−0.04–0.09	0.19	−0.27–0.32	0.19	−0.37–0.83	−0.87	−3.20–1.06
Femur length (percentile)	−0.04	−0.01–0.01	0.09	−0.02–0.02	0.20	−0.07–0.16	−0.13	−0.17–0.11
Estimated Birthweight (grams)	0.05	−0.01–0.01	0.75	−0.04–0.06	0.38	−0.02–0.09	−2.94	−0.53–0.03
Estimated Birthweight (percentile)	−0.30	−0.03–0.01	0.06	−0.04–0.05	−0.32	−0.30–0.10	−0.68	−0.48–0.05
Placental volume (mm^3^)	0.01	0.01	0.15	−0.01–0.01	0.46 *	0.01–0.08 *	0.57	0.02–0.09
VI	0.02	0.12	0.21	−0.03–0.08	−0.18	−1.00–0.36	−0.19	−1.12–0.46
FI	−0.01	−0.15	−0.12	−0.01–0.01	−0.11	−0.15–0.08	−0.11	−0.17–0.10
VFI	0.02	0.11	0.23	−0.04–0.13	−0.19	−1.54–0.51	−0.20	−1.71–0.67

BMI: body mass index; GA: gestational age; VI: Vascularization Index; FI: Flow Index, VFI: Vascularization Flow Index. * *p* < 0.05; ** *p* < 0.001.

## Data Availability

The data can be made available by the corresponding author on request.
